# Cracking the mystery of autism spectrum disorder

**DOI:** 10.1016/j.ebiom.2020.102691

**Published:** 2020-02-20

Globally, about 1 in 160 children is diagnosed with autism. The underlying pathology of autism spectrum disorder (ASD) remains unclear and no effective treatment is available. However, progress has been made in uncovering potential causative factors of autism and understanding how they might shape the pathogenesis of this disease.

ASD is generally believed to be caused by both genetic and environmental factors. A 2019 epidemiology study published in *JAMA Psychiatry* by Sandin (Karolinska Institute, Stockholm, Sweden) and collaborators estimated that genetic factors could contribute to up to 80% of ASD occurrence. Hence, identifying ASD-related genes and risk variants has been a major focus of ASD research. Empowered by advances in sequencing technology and the availability of large genomic datasets, many novel ASD risk variants have been identified, including de-novo gene mutations and common polygenic variants. De-novo gene mutations are estimated to account for about 30% of ASD cases. On Jan 23, a team led by Buxbaum (Aarhus University, Denmark) and Daly (Broad Institute, Cambridge, MA, USA) published a comprehensive exome sequencing study of 11 986 autistic patients in *Cell*. This study increased the number of high-confidence autism susceptibility genes to 102. Unlike de-novo mutations, the number of common genetic variants is huge, but each variant might individually only play a tiny role in the development of ASD. Polygenic variants are shown to account for a substantial part of ASD susceptibility and explain 17–52% of the heritability of the disease.

Environmental factors could also contribute to ASD. Increasing attention has been paid to events such as early-life exposure to environmental pollution, diabetic pregnancies, or prenatal infections. In particular, prolonged and repeated systemic immune activation and chronic inflammation have been proposed as environmental factors involved in ASD aetiopathology. As early as 2010, in an epidemiological analysis published in the *Journal of Autism and Developmental Disorders*, Atladóttir (University of Aarhus, Århus, Denmark) and colleagues suggested that severe viral infections during the first trimester of pregnancy increased autism risk by three times. Later studies of autistic patients have also suggested immune dysfunction as a potential contributing factor. Several proinflammatory cytokines, such as interleukin (IL)-6 and IL-17a, were found to be associated with ASD. Although the specific cellular targets and pathways in the brain are unclear, it is thought that IL-17a signalling during pregnancy might contribute to developmental defects in part of the somatosensory cortex called the S1DZ. In December, 2019, Choi and Huh's teams from MIT and Harvard Medical School (Boston, MA, USA) published an article in *Nature* that further underscores the role of the immune system in ASD.

Using a preclinical model of ASD in which mice display abnormal social interactions, the authors exposed the mice to lipopolysaccharide (LPS) to induce fever and an immune response. In response to LPS exposure, these mice released IL-17a and—surprisingly—displayed a temporary improvement in social behaviour. Further experiments revealed that IL-17a could bind to receptors in the S1DZ region and inhibit neural activity, thus substantially improving social interactions. IL-17a signalling in the SIDZ region might therefore have a dual effect, depending on the timing and context of IL-17a exposure. Another potentially intriguing implication of this article is that the findings could help to explain the connection between fever and symptomatic resolution in ASD. Bal and colleagues from University of California (San Francisco, CA, USA) reported that the behavioural symptoms of 362 (17%) of 2152 children were attenuated during fever (*Autism Research,* 2018). Although the IL-17a–SIDZ signalling pathways are yet to be confirmed in humans, IL-17a might be a molecular clue to the fever phenomenon. As the gut microbiome is known to play a role in IL-17 regulation, future studies will be aimed at exploring whether modulation of gut bacteria could have a similar beneficial effect.

Several genetic animal models have been developed to help advance the mechanistic understanding of ASD and direct gene-based therapies for it. These animal models are usually based on one or more known genetic variants of autism. Rodents have been the top choice for a long time, and although they provide much information about the molecular basis of disease, there are also disadvantages in using them to study neurodevelopmental disorders. In particular, mice do not have a highly developed prefrontal cortex—a unique feature of many primates, responsible for decision-making, maintaining concentration, and interpreting social cues. In a 2016 article in *Nature*, Zilong Qiu's team from Institute of Neuroscience, Chinese Academy of Sciences (Shanghai, China) published the first monkey model for Rett syndrome by overexpressing methyl-CpG binding protein 2, encoded by a gene that is associated with both Rett syndrome and ASD. In 2019, another study published in *Nature* by Zhou et al (South China Agricultural University, Guangzhou, China and Broad Institute Cambridge, MA, USA) reported the generation of a *SHANK3*-mutant macaque model for ASD research. These non-human primate models might be better suited than mouse models for studying the social aspects of human ASD.

Despite some encouraging growth in the field, we are still far from fully understanding the mechanistic and functional basis of ASD. The development of advanced techniques and improved preclinical models with translational insights will greatly help to move this field forward. EBioMedicine will continue to welcome cutting-edge autism studies that not only offer novel mechanistic insights but also contribute to the development of successful therapies.

*EBioMedicine*

